# Genetic analysis carried out in population tails reveals diverse two-loci interactions as a basic factor of quantitative traits variation in rye

**DOI:** 10.1007/s13353-015-0321-6

**Published:** 2015-10-08

**Authors:** Piotr Masojć, Anna Bienias, Marcin Berdzik, Piotr Kruszona

**Affiliations:** Department of Genetics, Plant Breeding and Biotechnology, West Pomeranian University of Technology in Szczecin, Słowackiego 17, 71-434 Szczecin, Poland

**Keywords:** *GA3ox*, Genetic variation, Quantitative traits, *Secale cereale* L, Two-loci interaction

## Abstract

Bidirectional selective genotyping carried out independently for five quantitative traits within a biparental population of recombinant inbred lines of rye has revealed dramatic changes in alleles distribution in the population tails. A given allele, predominant in the lower tail, is often neutral for reversely directed selection or associates with the upper tail following divergent selection for a related trait. Such radical changes in the alleles distribution cannot be explained by differences in genotypic values within a single locus. This paper presents the theoretical model of a genetic mechanism underlying observed responses of individual loci to divergent selection. The presented model refers to the specific interactions between alleles at two loci. Its wider application in genetic analysis will open up new possibilities for testing positions of genes in the hierarchical structure of interacting loci revealed under selection pressure.

## Introduction

Bidirectional selective genotyping (BSG) within a biparental population of recombinant inbred lines (RILs) of rye carried out on a genomic scale revealed complex, partially overlapping sets of loci related to preharvest sprouting (PHS), alpha-amylase activity (AA) (Masojć et al. 2009, 2011) and falling number (FN) (Masojć et al. not published). A trait-locus relationship was postulated when significant distortion of allelic segregation from the Mendelian 1:1 ratio was observed in any of the two groups of RILs with extreme phenotypes representing opposite population tails. The majority of detected molecular marker loci showed some segregation distortions only within one subset of RILs with extreme trait values. They were assigned to the R class when segregation distortion was found in a population tail related to the positive direction of selection. When segregation distortion was associated with the negative direction of selection, the loci were grouped into E class. The asymmetrical distribution of alleles in the population tails found in R and E classes reflects locus importance for one direction of selection and neutrality for the selection in the opposite direction.

A number of loci showing segregation distortions in both groups of extreme RILs, selected from population tails, were found in the case of PHS and AA. Loci representing symmetrical response to divergent selection, i.e. distorted segregation and predominance of different alleles in the opposite tails, were attributed to the D class (directional loci). Similar examples of distorted segregations were reported in rye for molecular marker loci responding to divergent selection for tolerance and susceptibility to reduced level of nitrogen and potassium in the growing medium (Smolik 2013). BSG analysis in rye revealed a number of loci associated with more than one trait. In spite of the fact that population tails contained different RILs for each trait, common markers often represented the same class irrespective to the trait studied. For some other marker loci their class appeared to be trait-specific. The accumulating data on various responses of individual loci to divergent selection raises a question on genetic basis of the observed differences.

This paper presents a theoretical model of genetic mechanism explaining the experimental results of different responses of molecular marker loci to divergent selection carried out for related traits of rye.

## Materials and methods

Two inbred lines of rye used in this study represent extremely different phenotypes with respect to several quantitative traits. Line 541 (more than 23 generations of inbreeding) is high, has long spikes and bending leaves. It is also highly susceptible to PHS, has elevated AA in the grain and, in addition, shows very low FN. Line Ot1-3 (more than 21 generations of inbreeding) is low, has short spikes and upright leaves. It is one of the most resistant lines with respect to PHS; has grain of low AA and high FN.

During the last decade, these traits have been investigated within individual projects aiming at identification of QTL and genes affecting their variation. The general method used was based on divergent selection for extreme trait values carried out within RILs populations of the 541×Ot1-3 biparental cross. Groups of RILs with extreme trait values selected from population tails were subjected to bidirectional selective genotyping. The first analysed trait was resistance to PHS. A selection for extreme PHS values was carried out, starting from large F_2_ generation (5000 plants), and was continued in each consecutive year up to F_7_ RILs progeny (Masojć et al. 2009). BSG was carried out on 20–30 RILs with high resistance to PHS (0-5 % sprouted kernels) and 20–30 RILs with high susceptibility (80-100 % sprouted kernels). In the course of this study a large number of selection responsive loci (SRL) were identified and mapped on all rye chromosomes. The search for loci, affecting variation of alpha-amylase activity, was based on population of 140 RILs (F_7–11_) from which two opposite extreme groups were selected. The first group showing extremely high AA (8.0-40.0 U/ml) included 14 RILs, and the second one with very low enzyme activity (0.0-0.2 U/ml) in the grain contained 14 RILs (Masojć et al. 2011). The same population was used to perform BSG for falling number. It was possible to select 19 RILs with high (180-340 s) and 17 RILs with the lowest possible FN (60s). The next trait being investigated was the leaf posture (LP) at the stage of heading. Out of 400 RILs of the F_6_-F_10_ generation, showing predominantly intermediate types (partially bending leaves), the two extreme groups were collected. The first contained 30 lines with upright leaves like in Ot1-3 line and the second included 30 lines with totally bending leaves as in 541 line. Divergent selection for spike length carried out within F_5–7_ generation of 300 RILs allowed to distinguish 20 RILs of short spike (6.5-8.5 cm) and 20 RILs of long spike (10.0-13.4 cm). Groups of RILs, with extreme phenotypes, were established on the basis of the data collected from at least three years.

DNA was isolated from leaves of young rye plants grown on the experimental field of West Pomeranian University of Technology in Szczecin, Poland. The leaves were immediately frozen, lyophilized (Christ Alpha1-2 LD plus) and ground into powder (Retsch MM200). DNA extraction was carried out using DNeasy Plant Mini Kit, Qiagen according to the manufacturer’s instructions. DNA concentration was measured in BioTek Epoch apparatus and equillibrated for analysis.

*GA3ox* gene in rye was amplified by using homologous sequences from wheat (DQ118252, DQ118250) and barley (AB189152) deposited in NCBI database. Primers were designed for wheat-barley consensus sequences. Monomorphic PCR products representing sequences of parental Ot1-3 and 541 lines were extracted from agarose gel using silicone columns (Qiagen). They were cloned using pCR2.1TOPO vectors in the presence of topoisomerase and *E. coli* strain transformed by the heat shock method (Invitrogen). Plasmids containing the insert were sequenced by Sanger method by means of a capillary sequencer CEQ 8000 (Beckman Coulter). BLAST analysis of rye sequences, carried out using nBLAST package available from the NCBI platform, proved their high homology (96-97 %, E = 2e-103) to wheat *GA3ox* sequence. In addition SNP and IN/DEL mutations differentiating parental lines of rye were found. These polymorphisms were used for the generation of allele specific markers (AS PCR) which were applied in this study. *GA3ox* gene was mapped on the proximal part of 3RL chromosome arm (unpublished data).

The remaining molecular markers were detected by the method of random amplified polymorphic DNA (RAPD) using one primer or a pair of arbitrary 10-mer primers (Williams et al. 1990; Masojć et al. 2001). Each RAPD marker was mapped on the rye genetic map (Milczarski et al. 2007; Masojć et al. 2009, 2011) which showed their single locus status and not distorted allelic segregation, according to Mendelian 3:1 (F_2_) or 1:1 (RILs) ratio.

Electrophoresis was carried out in 1.5 % agarose gels (1 × TBE buffer) at 5 V/cm for 10 min and then at 9 V/cm for 90 min using Bio-Rad PowerPac 300 power supply. Gels were incubated for 15 min in 0.1 mg/ml of ethidium bromide dissolved in TBE buffer. Electrophoregrams were visualized under UV light in a Syngene G:Box using a GeneSnap software.

A marker association with trait variation was established by finding statistically significant distortion from the Mendelian 1:1 segregation ratio within one or two groups of RILs selected for extreme trait values. The χ^2^ test at p $\le$ 0.05 was used for this purpose. Individual loci were classified according to the method described earlier (Masojć et al. 2009, 2011).

## Results

Nine molecular markers showing the most diversified patterns of association with the three or two of the five studied traits are presented in Table 1. Their comparative analysis reveals a genetic mechanism controlling alleles’ response to phenotypic selection. Almost each marker locus is assigned to a different class depending on its relationship with a phenotypic trait. Particular alleles are often associated with the opposite direction of selection for the two related traits. QTL represented by marker pr139_340 bp belongs to class D with respect to PHS. An allele originated from parental line Ot1-3, a source of resistance to preharvest sprouting, prevails in RILs with high resistance to PHS and in RILs of low FN. PHS resistance and low FN represent positive and negative traits for quality breeding, respectively and therefore marker-assisted selection, based on allele of line Ot1-3, would be ineffective. RILs of PHS susceptible group contain the predominant allele from easily sprouting parental line 541. The same allele is associated with extremely low AA. Again, one allele, originating from line 541, associates with negative (susceptibility to PHS) and positive (low AA) traits affecting grain quality. Similar examples of alleles associated with the opposite directions of selection for related traits are found in loci: pr310/320_650bp (PHS–AA, AA-FN), pr910_490bp (AA – FN), pr434/483_700bp (PHS–FN, AA–FN) and pr738a_1100bp (PHS-AA, PHS-FN). Different response to selection for related traits was also observed in loci: 884/873_280bp, pr611a_600bp and pr641_550bp. Here, each of the two alleles shows relationship or neutrality with respect to the trait depending on the direction of selection. Presented data were obtained through the analysis of molecular marker loci of unknown function representing QTL underlying variation of the studied traits.

**Table 1:** Different responses of molecular markers and genes loci to divergent selection for the highest and the lowest values of related quantitative traits in RILs progeny of rye intercross 541×Ot1-3

Locus, map location	Trait	Alleles segregation in group 1 representing positive direction of selection		Alleles segregation in group 2 representing negative direction of selection		Locus class	Origin of the pre- dominant allele in group 1	Origin of the pre- dominant allele in group 2
pr139_	PHS	Resistant	17 : 3**	Susceptible	5 : 15*	D	Ot1-3	541
340 bp	AA	Low	1 : 9*	High	5 : 6	R↺	541	-
5RL	FN	High	10 : 9	Low	12 : 4*	E↺	-	Ot1-3
pr310/320_	PHS	Resistant	11 : 9	Susceptible	4 : 16**	E	-	541
650 bp	AA	Low	2 : 9*	High	6 : 4	R↺	541	-
2RL	FN	High	9 : 10	Low	3 : 12*	E	-	541
pr910_	AA	Low	7 : 6	High	2 : 12**	E↺	-	Ot1-3
490bp, 2RS	FN	High	4 : 15*	Low	8 : 8	R	Ot1-3	-
pr434/483_	PHS	Resistant	4 : 14*	Susceptible	18 : 1**	D	Ot1-3	541
700 bp	AA	Low	3 : 11*	High	13 : 1**	D	Ot1-3	541
3RL	FN	High	14 : 4*	Low	9 : 6	R↺	541	-
pr738a_	PHS	Resistant	8 : 12	Susceptible	16 : 4**	E	-	541
1100 bp	AA	Low	10 : 1**	High	5 : 5	R↺	541	-
7RL	FN	High	14 : 5*	Low	7 : 9	R↺	541	-
pr884/873_	PHS	Resistant	16 : 4**	Susceptible	10 : 10	R	Ot1-3	-
289 bp 1RL	FN	High	11 : 8	Low	4 : 13*	E	-	541
pr611a_	PHS	Resistant	17: 2**	Susceptible	8 : 12	R	Ot1-3	-
600 bp	AA	Low	5 : 4	High	1 : 10**	E	-	541
1RL	FN	High	10 : 9	Low	12 : 4*	E	-	541
pr641_	PHS	Resistant	4 : 14*	Susceptible	11 : 9	R	Ot1-3	-
550 bp	AA	Low	6 : 7	High	11 : 3*	E	-	541
6RL	FN	High	8 : 11	Low	2 : 14**	E	-	541
*GA3ox*_	PHS	Resistant	16 : 4**	Susceptible	4 : 16**	D	Ot1-3	541
250 bp	SL	Long	9 : 11	Short	5 : 15*	E↺	-	541
3RL	LP	Upright	8 : 22**	Bending	16 : 14	R↺	541	-

PHS—preharvest sprouting, AA—alpha-amylase activity, FN—falling number, SL—spike length, LP—leaf posture

* Distorted segregation—significant deviation from the 1:1 ratio according to χ^2^ test at p ≤ 0.05 (*) or at p ≤0.01 (**)

↺ Reversed class—allele selected to the opposite group of RILs with extreme trait values

Similar differences in alleles distribution between population tails were found for *GA3ox* gene encoding GA3 oxidase, a key enzyme in the production of bioactive forms of giberrellic acid (GA_4_/GA_1_) in plants (Table 1). *GA3ox* shows pleiotropic effects for traits associated with GA production, i.e. PHS, SL and LP. Alleles of *GA3ox* exhibit trait specific distribution within population subsets of extreme phenotypes. *GA3ox* locus belongs to class D with respect to PHS and seems to be one of the most important loci controlling sprouting resistance. Allele originating from line Ot1-3 is associated with resistance to PHS and allele derived from line 541 shows a relationship with susceptibility to sprouting. The same *GA3ox* locus controls spike length but for this trait it represents class E↺, i.e. reversed class E, since *GA3ox* allele of parental line 541 having long spike prevails in RILs representing lower population tail showing association with development of a short spike. The third function of *GA3ox* gene revealed through BSG study is the control of leaf development. Analysis of RILs with extremely different leaf posture (upright vs. bending leaves) shows that during plant vegetative growth, an allele of line 541, which has bending leaves, associates with upright leaves and thus represents R↺ class (R reversed class) for this trait. *GA3ox* gene showed no relationship with alpha-amylase activity and falling number and therefore it is of 0 class for both these traits.

**Fig. 1:**
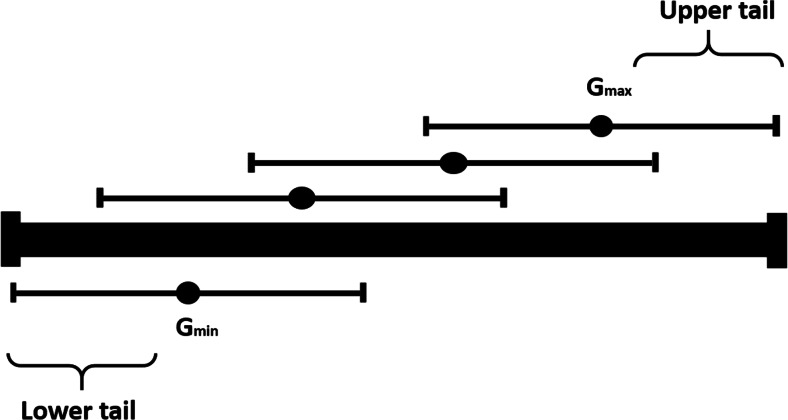
A theoretical example of the possible relationship between genotypic values (G_x.y_) and variation ranges of the four double homozygotes relative to variation range of the quantitative trait in a biparental population of RILs. Due to the shift of variation ranges preferably genotypes of G_min_ and G_max_ will be collected at the lower and upper population tails respectively as described in the model (Table 2)

Different patterns of alleles distribution in population tails presented in Table 1 cannot be explained by intra-locus mechanisms since there is no dominance effect in a RILs population. They may rather reflect interaction of at least two polymorphic loci taking place on DNA, mRNA, the protein or metabolic levels and affecting trait variation. The genetic consequences of such interaction are considered in the model presented in Tables 2 and 3 and in Fig. 1. The two interacting loci were given names *Exp* and *Res* since their joint expression defines activity/amount of enzyme(s)/protein(s) or metabolite(s) involved in the processes affecting trait’s value. *Exp* and *Res* genes may represent regulatory and structural loci, two regulatory loci or two structural loci encoding two enzymes or enzyme and its inhibitor. The model also includes miRNA and other polymorphic RNA loci which may interfere with gene expression. In a biparental population of homozygotes showing wide variation range with respect to the quantitative trait a pair of interacting loci may generate four genotypes with different genotypic values (G_x.y_) being a reason for trait variation. Two of these genotypes representing the two extreme genotypic values, i.e. the lowest (G_min_) and the highest (G_max_) outnumber genotypes with intermediate G values (G_int1_ and G_int2_) within the most distant intervals of population (population tails) when differences between G_int1_ and G_min_ and between G_max_ and G_int2_ are significant (Table 2, Fig. 1). Alleles’ segregation with statistically significant distortion from the Mendelian 1:1 ratio observed within a subset of RILs with extreme trait values is proof of substantial differences between appropriate G values (Tables 1 and 3). When the difference between G_max_ and G_int2_ or between G_int1_ and G_min_ values is below a threshold (assumed to be 5 % for the model examples), both genotypes are found in the population tail with similar frequencies giving alleles’ segregation according to Mendelian 1:1 ratio in one of the two loci. Simulation of the relationship between the d values and the ratio between frequencies of genotypes with G_max_ and G_int2_ genotypic values has been made assuming that the standard curve of normal distribution describes variation of the studied trait (Table 4). It shows that with the increase of the d value the rate of the genotypes’ frequencies within the population upper tail increases from the values mainly below 2 (d = 3.0 % ) up to 10.5 (d = 10 %). For each level of the d value, the highest ratio of the two genotypes frequencies is exhibited at 2.5 % and the lowest at the 10 % selection rate. The χ2 test performed within population tails to assess significance of deviation from the 1:1 alleles’ Mendelian segregation shows that the ratio close to 3:1 is the usual threshold level (Table 1). It is achieved for d = 5 % and d = 7.5 % at the 2.5 % selection rate but with lower probability significant results can also be obtained at the 5.0 % selection rate. With the increase of the difference between genotypic values up to 10.0 % it can be confirmed by genetic analysis within population tails at 5.0 % and 10.0 % selection rates. It means that the population size of 300 RILs should be sufficient to detect 10 % differences in genotypic values by performing the χ^2^ test on 30 individuals with extreme phenotypes. The presented simulation shows that choosing the threshold of 5.0 % for the d value in the analysed two-loci model is justified since it is the lowest d value giving significant values in the χ2 test at the 2.5 % selection rate.

**Table 2:** A model of interaction between *Exp* and *Res* loci, explaining observed alleles distribution in tails of RILs population. Four homozygous genotypes within biparental population representing advanced generation of inbreeding attain various genotypic values (G_x.y_) as a result of different combinations of *Exp* and *Res* alleles

Locus/alleles	*Res*_*1*_ /	*Res*_*2*_ /
*Exp*_*1*_ /	*Exp*_*1*_ // *Exp*_*1*_, *Res*_*1*_ // *Res*_*1*_ **G**_**1.1**_	*Exp*_*1*_ // *Exp*_*1*,_ *Res*_*2*_ // *Res*_*2*_, **G**_**1.2**_
*Exp*_*2*_ /	*Exp*_*2*_ // *Exp*_*2*_, *Res*_*1*_ // *Res*_*1*_ **G**_**2.1**_	*Exp*_*2*_ // *Exp*_*2*_, *Res*_*2*_ // *Res*_*2*_ **G**_**2.2**_

where:

1. **G**_**x.y**_ (%) are genotypic values of double homozygotes (means of their phenotypic values), relative to the highest trait value detected in the RILs population

2. G_2.2_ ≫ G_1.1_—an assumption resulting from the higher genotypic value of parental line 2 relative to that of line 1

3. The four G_x.y_ values are distributed within the population variation range in the following order:

G_min_ < G_intermediate1_ < G_intermediate2_ < G_max_

The extreme genotypes will be overrepresented in population tails relative to the intermediate ones causing alleles’ segregation distortions from the Mendelian 1:1 ratio, when differences (d) between G_max_ and G_intermediate2_ (G_int2_) or between G_intermediate1_ (G_int1_) and G_min_ values are above a certain threshold level. The d value of 5 % is assumed to be the least significant in this theoretical model. Depending on the distribution of the d values below a threshold level there may be six different results of divergent selection:

a) Genotype of the G_min_ value will be overrepresented within the lower tail and genotype of the G_max_ value will be overrepresented within the upper tail, when G_int1_-G_min_ ≥ 5 %, G_max_-G_int2_ ≥ 5 %

b) Genotypes of G_min_ and G_int1_ values will be present in similar frequencies within the lower tail and genotype of G_max_ value will be overrepresented within the upper tail, when G_int1_-G_min_ < 5 %, G_max_-G_int2_ ≥ 5 %

c) Genotype of G_min_ value will be overrepresented within the lower tail and genotypes of G_max_ and G_int2_ values will be present in similar frequencies within the upper tail, when G_int1_-G_min_ ≥ 5 %, G_max_-G_int2_ < 5 %

d) Genotypes of G_int1_ and G_min_ values will be present in similar frequencies within the lower tail and genotypes of G_max_ and G_int2_ values will be present in similar frequencies within the upper tail, when G_int1_-G_min_ < 5 % and G_max_-G_int2_ < 5 %

e) Genotypes of G_min_, G_int1_ and G_int2_ values will be present in similar frequencies within the lower tail and genotype of G_max_ value will be overrepresented within the upper tail, when G_int2_-G_int1_ < 5 %, G_int2_-G_min_ < 5 %, G_int1_-G_min_ < 5 % and G_max_-G_int2_ ≥ 5 %

f) Genotype of G_min_ value will be overrepresented within the lower tail and genotypes of G_int1_, G_int2_, G_max_ will be present in similar frequencies within the upper tail, when G_int1_-G_min_ ≥ 5 % and G_max_-G_int2_ < 5 %, G_max_- G_int1_ < 5 %, G_int2_-G_int1_ < 5 %.

4. Variation of a quantitative trait can be controlled by a number of pairs of interacting loci. The most extreme values of the quantitative trait detected in population tails result from pyramiding of favorable alleles from many interacting loci in a particular genotype

**Table 3:** The theoretical examples of two-loci interaction resulting in different alleles’ distribution in groups of RILs assembled through divergent selection for quantitative trait, carried out within biparental population. It is assumed that d = 5 % is the lowest significant difference between genotypic values (G_x.y_). The predominance of one allele or segregation of alleles, according to the Mendelian ratios (1:1 or 2:1), are observed within the selected groups depending on differences between the genotypic values. G_x.y_ values of genotypes which will be found with high frequency within the lower and upper tails are in bold and in bold italics, respectively

No	Genotypic values (%) resulting from the combined action of *Exp* and *Res* alleles			Alleles segregation within extreme RILs with desirable phenotype (lower tail)		Alleles segregation within extreme RILs with undesirable phenotype (upper tail)		Class of selection responsive locus, ↺-allele selected to the opposite extreme group (reversed class)	
	Allele	*Res*_*1*_	*Res*_*2*_	*Exp* locus	*Res* locus	*Exp* locus	*Res* locus	*Exp* locus	*Res* locus
1	*Exp*_*1*_	**40**	***56***	*1Exp*_*1*_ : *1Exp*_*2*_	*Res*_*1*_	*1Exp*_*1*_ : *1Exp*_*2*_	*Res*_*2*_	0	D
	*Exp*_*2*_	**44**	***60***						
2	*Exp*_*1*_	**40**	54	*Exp*_*1*_	*Res*_*1*_	*Exp*_*2*_	*Res*_*2*_	D	D
	*Exp*_*2*_	46	***60***						
3	*Exp*_*1*_	**39**	56	*1Exp*_*1*_ : *1Exp*_*2*_	*Res*_*1*_	*Exp*_*2*_	*Res*_*2*_	E	D
	*Exp*_*2*_	**43**	***62***						
4	*Exp*_*1*_	**36**	***59***	*Exp*_*1*_	*Res*_*1*_	*1Exp*_*1*_ : *1Exp*_*2*_	*Res*_*2*_	R	D
	*Exp*_*2*_	43	***62***						
5	*Exp*_*1*_	**39**	55	*1Exp*_*1*_ : *1Exp*_*2*_	*Res*_*1*_	*Exp*_*1*_	*Res*_*2*_	E↺	D
	*Exp*_*2*_	**42**	***64***						
6	*Exp*_*1*_	48	***62***	*Exp*_*2*_	*Res*_*1*_	*Exp*_*1*_	*Res*_*2*_	D↺	D
	*Exp*_*2*_	**41**	49						
7	*Exp*_*1*_	42	***60***	*Exp*_*2*_	*Res*_*1*_	*1Exp*_*1*_ : *1Exp*_*2*_	*Res*_*2*_	R↺	D
	*Exp*_*2*_	**35**	***63***						
8	*Exp*_*1*_	**40**	**37**	*Exp*_*1*_	*1Res*_*1*_:*1Res*_*2*_	*Exp*_*2*_	*Res*_*2*_	D	E
	*Exp*_*2*_	58	***65***						
9	*Exp*_*1*_	45	**35**	*Exp*_*1*_	*Res*_*2*_	*Exp*_*2*_	*Res*_*2*_	D	F
	*Exp*_*2*_	55	***65***						
10	*Exp*_*1*_	**35**	45	*Exp*_*1*_	*Res*_*1*_	*Exp*_*2*_	*Res*_*1*_	D	F
	*Exp*_*2*_	***65***	55						
11	*Exp*_*1*_	**35**	***65***	*Exp*_*1*_	*Res*_*1*_	*Exp*_*1*_	*Res*_*2*_	F	D
	*Exp*_*2*_	45	55						
12	*Exp*_*1*_	45	55	*Exp*_*2*_	*Res*_*1*_	*Exp*_*2*_	*Res*_*2*_	F	D
	*Exp*_*2*_	**35**	***65***						
13	*Exp*_*1*_	**39**	48	*Exp*_*1*_	*Res*_*1*_	*Exp*_*2*_	*1Res*_*1*_ : *1Res*_*2*_	D	R
	*Exp*_*2*_	***55***	***58***						
14	*Exp*_*1*_	**38**	**40**	*Exp*_*1*_	*1Res*_*1*_:*1Res*_*2*_	*Exp*_*2*_	*1Res*_*1*_ : *1Res*_*2*_	D	0
	*Exp*_*2*_	***59***	***63***						
15	*Exp*_*1*_	**45**	**48**	*2Exp*_*1*_ : *1Exp*_*2*_	*2Res*_*1*_:*1Res*_*2*_	*Exp*_*2*_	*Res*_*2*_	E*	E*
	*Exp*_*2*_	**46**	***61***						
16	*Exp*_*1*_	**37**	***54***	*Exp*_*1*_	*Res*_*1*_	*1Exp*_*1*_ : *2Exp*_*2*_	*1Res*_*1*_ : *2Res*_*2*_	R*	R*
	*Exp*_*2*_	***53***	***56***						
17	*Exp*_*1*_	**40**	**43**	*Exp*_*1*_	*1Res*_*1*_:*1Res*_*2*_	*Exp*_*2*_	*Res*_*1*_	D	E↺
	*Exp*_*2*_	***62***	55						
18	*Exp*_*1*_	60	**35**	*Exp*_*1*_	*Res*_*2*_	*Exp*_*2*_	*1Res*_*1*_:*1Res*_*2*_	D	R↺
	*Exp*_*2*_	***51***	***54***						
19	*Exp*_*1*_	46	**44**	*Exp*_*2*_	*Res*_*2*_	*Exp*_*2*_	*Res*_*1*_	D	D↺
	*Exp*_*2*_	***62***	48

**Table 4:** Simulation of the frequencies ratio of the two double homozygous genotypes having G_max_ and G_int2_ genotypic values in the upper tail of biparental RILs population depending on differences between their G values (d) and selection rate. Estimations were made using the curve of normal distribution. Tolerance of 5 % for the tails’ cut off lines was allowed

d— difference between G values	Selection rate 2.5 %	5.0 %	10.0 %	Effect of divergent selection (χ^2^ test)
3 %	1.8-2.0	1.6-1.9	1.6-2.0	Not significant at any of the tested selection rates
5 %	2.7-4.2*	2.1-3.2	1.9-2.2	Significant at 2.5 % selection rate
7.5 %	3.5-5.5*	2.5-3.1	2.3-2.5	Significant at 2.5 % selection rate
10 %	7.6-10.5*	4.7-5.7*	3.5-4.0*	Significant at each assessed selection rate
Population size	1200	600	300	30 extreme RILs from each tail

Table 3 presents simulation of relationship between various possible distributions of significant and insignificant d values among two-loci genotypes and resulting patterns of alleles’ distribution in population tails being a basis for individual loci classification. It is assumed that a threshold for the significant d value (difference between G values) is 5 % and mean trait value (population mean) is 50 %. In real experiments the threshold for the d value depends on population size and selection intensity (the higher population size and more intense selection the lower the d threshold). In complex multi-loci traits genotypic values calculated for two-loci genotypes do not deviate much from population mean and therefore G values in the model examples oscillate not more than 15 % from the population mean. As shown in Table 3, there is a wide range of possible patterns of alleles distribution in population tails depending on the differences in genotypic values of four genotypes within the two interacting loci. When two extreme G values are represented by two separate genotypes, loci interaction types: D-D, D-D↺, D↺-D, F-D or D-F are observed. When one of the extreme G values is attained by two genotypes and second by one genotype the following types of interaction: D-R, R-D, D-R↺, R↺-D, D-E, D-E↺ are possible. In the case when the level of extreme G value is reached by two genotypes, the two loci interact according to D-0 and 0-D schemes, where 0 designates a selectively neutral class. When one extreme G value is represented by one genotype and another is reached by three remaining genotypes, interaction types R*-R* and E*-E* are found, and alleles segregation according to 2:1 or 1:2 ratio is observed. There is a certain relationship between the classes of *Exp* and *Res* loci in the presented model. When *Exp* locus belongs to the D class, the *Res* locus can represent any class, i.e. D, E, E↺, R, R↺, F or 0 and *vice versa*. In the case of R, E or F class in one locus, the second interacting locus must be of class D. The most surprising pattern of the alleles distribution in population tails is represented by the F class (fixed allele). The same allele is predominant in both tails of the population. This is possible on the condition that specific homozygote at the locus of F class shows the highest and the lowest genotypic values in an interaction with the first and the second alleles of the D class locus, respectively. Interaction types: R-R, E-E, E-R, F-R, F-E, R-R↺, E-E↺, E-R↺, R-E↺ and R-0, E-0 are not possible according to the presented two-loci model.

The model includes epistatic and non-epistatic relationship between the two loci. In the case of D-D, D-D↺ and D↺-D interaction types, the two loci may affect the studied trait independently from each other in the additive way as they may contribute to the trait value being active in different developmental pathways. Alternatively, two-loci interaction according to D-D, D-D↺ and D↺-D models may result from the loci relationship within the same developmental pathway. Such interaction may exert additive or epistatic effects on the trait variation. All other inter-loci relationships shown in Table 3 represent epistatic interactions, where allele of the D class locus (epistatic) modify phenotypic effects of alleles at the E, R or F class (hypostatic loci). In the D–E interaction type, positively acting allele of the D locus suppresses or makes indifferent expression of both alleles from the E locus while negatively acting allele of the D locus enhances or sustains differential expression of alleles in the E locus. In the D–R interaction type positively acting allele of the D locus promotes different expression of alleles in the R locus and negatively acting allele of the D locus suppresses or makes indifferent expression of both alleles at the R locus.

D-0 type of interaction may have different genetic background. The simplest explanation assumes that the locus of class 0 is monomorphic and therefore it shows no allele segregation but the differential regulation of its expression is entirely dependent on a polymorphic D class locus. Theoretically, D-0 may also represent epistatic relationship when both polymorphic alleles of the D class increase or reduce the expression of both (possibly polymorphic) alleles at the hypostatic locus to the same level, thus making it of class 0. In this case the locus of class D should interact with other loci affecting the studied trait.

Experimental data (Table 1) correspond to the specified model examples (Table 3). Almost all classes of the interacting loci were found for the studied traits except D↺, F, E* and R*. It may mean that they represent rare types of two-loci interaction. According to the model, alleles distribution in the tails of population is directly determined by the relationship between G_x.y_ values. This relationship may be specific for different physiological or developmental processes that often take place at a defined time and specific cells of plant organs. The relationship among G values may also be different in separate crosses due to the involvement of other alleles. Therefore, a locus class may not be kept across traits and different biparental populations. In fact we found only one marker locus among many detected earlier (pr568 on 5RL) which represented the same E class for PHS, AA and FN. The results shown in Table 1 fully support conclusions drawn from the model examples that a class of locus depends on the trait studied and on the functional polymorphisms within RILs population. Locus pr139_340bp representing D class for PHS must be linked to important QTL as it may show any possible types of interaction with other loci. The same locus represents R↺ class for alpha-amylase activity and as such must interact according to the D-R↺ scheme (Table 3). The involvement of the same locus in control of falling number (E↺ class) should follow the D-E↺ model of interaction. *GA3ox* locus represents D class for PHS and can show any type of interaction. Its interaction type for spike length (E↺ class) should follow the D-E↺ scheme and for leaf posture (R↺ class) the D-R↺ model would be appropriate (Tables 1 and 3).

The second assumption included in the model (Table 2) is valid for most of the crosses and pairs of interacting loci. However, a low frequency of odds with G1.1 ≫ G2.2 is possible for complex quantitative traits. When G values for *Exp1*//*Exp1*, *Res1*//*Res1* and *Exp2*//*Exp2*, *Res2*//*Res2* genotypes are replaced, another series of model examples for unique situations can be generated. They will be a mirror reflection of those shown in Table 3, i.e. D-0 will be replaced by 0-D↺, D-D by D↺-D↺, D-E by E↺-D↺ and so on.

The suggested model relies on the interaction between the two polymorphic loci, assuming that possible additionally interacting loci are homozygous. However, the functional polymorphisms in three or more interacting loci, from the same developmental pathway within one biparental population, cannot be excluded. A three-loci model demands larger population size for the discrimination of G values among eight genotypes. It will generate predominantly R*, E* but also D, R, E classes known from the two-loci model. There will also be new common classes with alleles segregating in 1:1 ↔2:1 and 2:1 ↔2:1 ratios within the opposite population tails. Since classes of loci specific for a three-loci model were not found in the experimental data, a two-locus model seems to be more suitable for the studied RILs populations.

The model is well adjusted to genotypes where interacting loci are not linked or show a very loose linkage. However, it might be suspected that two tightly linked genes from different developmental pathways of the studied trait may both show functional polymorphism in a given biparental population. Such coincidence can negatively affect classification of loci and interaction types. So far the results showed that groups of loosely linked loci have often represented the same R or E class which suggests that the interaction with a common locus of the D class and not the linkage may be the main mechanism underlying such uniformity (Masojć et al. 2009, 2011). There are also some examples of two tightly linked loci with D and E class, indicating that the intensity of selection may often surpass linkage drag. Further studies should clarify to what extent the linkage can obstruct the detection of interactive loci.

## Discussion

In spite of the fact that bidirectional selective genotyping is applied in many other published studies, the problem of different classes of loci that can be discerned based on diverse patterns of alleles distribution between the tails of population variation was not addressed (Jerez-Timaure et al. 2005; Gallais et al. 2007; Sun et al. 2010; Vikram et al. 2012; Eskandari et al. 2013; Farkhari et al. 2013). The first report on the recognition of the three classes of loci (D, R, E), regarding their response to divergent selection for PHS, was published by Masojć et al. (2009). The loci underlying PHS were identified on each rye chromosome with a number of loci representing the D class accumulated in definite regions of 1RL, 3RL and 5RL. Loci of R class were found in specific regions on chromosome arms 1RL, 2RS, 6RL. A number of PHS loci of E class were localized mainly on chromosome arms 2RL, 5RL and 7RL. It was suggested that extreme trait values result from accumulation of positively acting alleles in loci of D and R classes (lower tail) and negatively acting alleles in loci of D and E classes (upper tail).

The second report on detecting of the three classes of selection responsive loci was published by Masojć et al. (2011) for alpha-amylase activity in rye grain. A complex set of loci dispersed over all rye chromosomes in regions similar to those related to PHS was identified with many examples of E and R classes and 2–4 cases of the D class. Additional evidence for existence of the three patterns of alleles distribution between population tails was submitted by Smolik (2013). Two rye biparental populations of RILs were tested by BSG method for nutrition deficiency stress. These experiments not only proved the existence of the three classes of loci controlling complex quantitative traits but also showed that the same locus responds to selection in a different way within the two compared intercrosses. The results of our unpublished BSG studies on falling number and leaf posture in rye show that the majority of detected SRL represent R or E classes which suggests that a vast part of quantitative trait variation results from the epistatic interactions. Similar results on high number of epistatic interactions were revealed for yield-related traits in rice (Xing et al. 2002). There is high probability that epistatic interactions contribute, in major part, to genetic variation within other eukaryotic species since a complex regulatory network functions in genomes of all living organisms.

Instances of allele prevalence in the opposite population tail relative to that represented by parental line, i.e. reversed classes are quite common for AA but not for PHS (Masojć et al. 2009, 2011). In total 16 AA loci of R↺ class mapped on chromosomes 2R, 4R, 5R and 7R were found with allele of line 541 (high AA) prevailing in RILs with extremely low alpha-amylase activity. These loci must represent R↺-D interaction type and therefore molecular marker loci of the D class detected mainly on chromosome 3RL and 2RS are candidates for epistatic genes that may have strong impact on alpha-amylase activity (Masojć et al. 2011). All AA loci of R↺, R, E, E↺ and D classes are mapped in distant positions from alpha-amylase structural genes located on chromosome arms 6RL and 7RL (Milczarski et al. 2007). This observation leads to the conclusion that all detected QTL for alpha-amylase activity belong to regulatory network. This regulatory network affects expression of a multi-gene family of alpha-amylase structural loci that do not show functional polymorphisms in the studied cross. Complex regulatory network for alpha-amylase activity in rye grain may be partially ascribed to the complexity of physiological processes leading to alpha-amylase synthesis in cereal grain (Mares and Mrva 2008; Mrva and Mares 2002).

The presented model defines instances when polymorphism in only one locus is sufficient to generate trait variation. Such cases are explained by D-0 interaction type where the lack of functional polymorphism in one interacting locus (class 0) is combined with polymorphism in a second locus of the D class. Single locus of the D class may be epistatic for a number of hypostatic loci of R and/or E classes and therefore it may affect trait variation to much more of an extent than hypostatic loci. Locus of the D class may explain a vast part of the trait variation when a mutant allele represents mutation suppressing all hypostatic loci. This is because the epistatic mutant allele in homozygous state may mask all functional polymorphisms that are generated by hypostatic loci. Consequently, loci of the D class seem to be the most valuable for breeding purposes. Their identification is an important step in developing strategy of efficient marker assisted selection. A complete strategy should also include knowledge of positive two-loci interaction of the D–R type and of pleiotropic effects within the breeding material.

Different distribution of *GA3ox* alleles in groups of RILs with extreme phenotypes for PHS, SL and LP detected in this study may result from their interaction with different regulatory genes active in particular developmental processes. This hypothesis is in agreement with data on *GA3ox* regulation in Arabidopsis (Curaba et al. 2004; Matsushita et al. 2007). *GA3ox* promoter contains *RY* and *CIS* regulatory elements specific for FUS3/LEC2 and AGF1 transcription factors expressed during seed maturation and plant growth, respectively. As presented in the model, the allele distribution in population tails depends on the two-loci genotypes and not on genotype at the single locus. A given allele can associate with the low and with the high trait value following the change of interaction just like *GA3ox* allele from line 541. Since the role of an individual allele in trait variation is relative and depends on the interaction with other loci, it is important to develop efficient methods of identifying pairs of interacting loci and alleles combinations exerting the strongest positive effects.

The main concern in BSG study is collection of the sufficient number of RILs representing extreme trait values. It is suggested that ca. 30 lines per each population tail should be enough for identification of QTL with a satisfying level of confidence (Farkhari et al. 2013). Then a population size of 300–400 RILs would represent a good compromise between demands of experiment accuracy and economy (Vikram et al. 2012). The simulation study presented in Table 4 suggests that use of 300–400 RILs allows to detect genotypic differences in BSG study starting from the 7.5-10.0 % level at the 10.0 % selection ratio.

The three reports on alleles segregation within population tails (Masojć et al. 2009, 2011 and this paper) indicate that the presented method of loci classification opens up new possibilities in analysis of genes interaction being a source of quantitative trait variation. We suggest a name genes interaction assorting by divergent selection (GIABDS) for this method. GIABDS coupled with genetic mapping allows for a genome-wide search for valuable genes and genes interactions. The identification of the trait-allele associations through identification of D, R and E classes of loci and various types of two-loci interactions seems to be an important step in revealing complex regulatory networks affecting quantitative traits. Loci interaction might be proven by using analytical tools of QTL mapping (Wang et al. 1999) and by determination of the G values through phenotyping and genotyping of each RIL from the developed biparental populations (Tranquilli and Dubcovsky 2000). This strategy needs integrated GIABDS and omics studies on well characterized large polymorphic populations including selected groups of RILs with extreme phenotypes.
